# Neighborhood-Level Socioeconomic Status and Prescription Fill Patterns Among Patients With Heart Failure

**DOI:** 10.1001/jamanetworkopen.2023.47519

**Published:** 2023-12-14

**Authors:** Amrita Mukhopadhyay, Saul Blecker, Xiyue Li, Ian M. Kronish, Rumi Chunara, Yaguang Zheng, Steven Lawrence, John A. Dodson, Sam Kozloff, Samrachana Adhikari

**Affiliations:** 1 Division of Cardiology, Department of Medicine, NYU Grossman School of Medicine, New York, New York; 2 Department of Population Health, NYU Grossman School of Medicine, New York, New York; 3 Department of Medicine, NYU Grossman School of Medicine, New York, New York; 4 Center for Behavioral Cardiovascular Health, Columbia University Irving Medical Center, New York, New York; 5 Department of Biostatistics, NYU School of Global Public Health, New York, New York; 6 Department of Computer Science & Engineering, Tandon School of Engineering, New York, New York; 7 NYU Rory Meyers College of Nursing, New York, New York; 8 Department of Medicine, University of Utah, Salt Lake City

## Abstract

**Importance:**

Medication nonadherence is common among patients with heart failure with reduced ejection fraction (HFrEF) and can lead to increased hospitalization and mortality. Patients living in socioeconomically disadvantaged areas may be at greater risk for medication nonadherence due to barriers such as lower access to transportation or pharmacies.

**Objective:**

To examine the association between neighborhood-level socioeconomic status (nSES) and medication nonadherence among patients with HFrEF and to assess the mediating roles of access to transportation, walkability, and pharmacy density.

**Design, Setting, and Participants:**

This retrospective cohort study was conducted between June 30, 2020, and December 31, 2021, at a large health system based primarily in New York City and surrounding areas. Adult patients with a diagnosis of HF, reduced EF on echocardiogram, and a prescription of at least 1 guideline-directed medical therapy (GDMT) for HFrEF were included.

**Exposure:**

Patient addresses were geocoded, and nSES was calculated using the Agency for Healthcare Research and Quality SES index, which combines census-tract level measures of poverty, rent burden, unemployment, crowding, home value, and education, with higher values indicating higher nSES.

**Main Outcomes and Measures:**

Medication nonadherence was obtained through linkage of health record prescription data with pharmacy fill data and was defined as proportion of days covered (PDC) of less than 80% over 6 months, averaged across GDMT medications.

**Results:**

Among 6247 patients, the mean (SD) age was 73 (14) years, and majority were male (4340 [69.5%]). There were 1011 (16.2%) Black participants, 735 (11.8%) Hispanic/Latinx participants, and 3929 (62.9%) White participants. Patients in lower nSES areas had higher rates of nonadherence, ranging from 51.7% in the lowest quartile (731 of 1086 participants) to 40.0% in the highest quartile (563 of 1086 participants) (*P* < .001). In adjusted analysis, patients living in the lower 2 nSES quartiles had significantly higher odds of nonadherence when compared with patients living in the highest nSES quartile (quartile 1: odds ratio [OR], 1.57 [95% CI, 1.35-1.83]; quartile 2: OR, 1.35 [95% CI, 1.16-1.56]). No mediation by access to transportation and pharmacy density was found, but a small amount of mediation by neighborhood walkability was observed.

**Conclusions and Relevance:**

In this retrospective cohort study of patients with HFrEF, living in a lower nSES area was associated with higher rates of GDMT nonadherence. These findings highlight the importance of considering neighborhood-level disparities when developing approaches to improve medication adherence.

## Key Points

**Question:**

Is living in a neighborhood with low neighborhood-level socioeconomic status (nSES) associated with increased nonadherence to life-saving medications for heart failure?

**Findings:**

In this cohort study of 6247 patients with heart failure with reduced ejection fraction, patients living in neighborhoods with lower nSES had significantly higher odds of nonadherence to guideline-directed medical therapy.

**Meaning:**

These findings suggest that interventions to improve medication adherence among patients with heart failure should consider neighborhood-level approaches and policies to target patients at highest risk.

## Teaser

This cohort study examines the association between neighborhood-level socioeconomic status and medication nonadherence among patients with heart failure (HF).

## Introduction

Treatment for heart failure with reduced ejection fraction (HFrEF) includes guideline-directed medical therapy (GDMT)^1^ with 4 categories: (1) β-blocker (BB); 2) angiotensin-converting enzyme-inhibitor, angiotensin receptor blocker, or angiotensin receptor neprilysin inhibitor (ACEI/ARB/ARNI); 3) mineralocorticoid receptor antagonist (MRA); and 4) sodium-glucose cotransporter-2-inhibitor (SGLT2i). GDMT has improved clinical outcomes in multiple trials,^1^ and contemporary analysis estimates a 4-fold decrease in mortality with this regimen.^2^ Despite substantial benefits, up to 40% to 50% of patients do not regularly fill these medications,^3,4,5^ leading to increased hospitalization and mortality.^6^

Neighborhood characteristics and the built environment (ie, the physical makeup of a community) may play a meaningful role in a patient’s ability to fill medications. For example, a patient may have difficulty filling prescriptions if they have inadequate access to transportation, are unable to walk to the pharmacy, or do not have pharmacies nearby. Low neighborhood socioeconomic status (nSES) has been associated with medication nonadherence in other clinical contexts, such as diabetes,^7^ chronic kidney disease,^8^ and cardiovascular disease prevention.^9^ Patients with HFrEF may be especially vulnerable to these effects given the high complexity of HF medication regimens^10^ and the frequent titration of GDMT doses and pills.^11^ Moreover, nSES may be associated with individual-level SES factors, such as income or medication cost, both of which have been associated with nonadherence to GDMT for HFrEF.^12,13,14,15^ Because individual-level SES information may not always be readily available, identifying neighborhood-level risk factors for medication nonadherence could play a key role in informing community-based and policy-level interventions to improve medication adherence. Therefore, we aimed to assess the association between nSES and medication nonadherence to GDMT for patients with HFrEF. Furthermore, given known associations between nSES and a neighborhood’s built environment, we examined the potential for mediation by built environment characteristics that could impact a patient’s ability to physically obtain their medications: access to transportation, walkability, and pharmacy density.^16,17,18^

## Methods

### Study Design, Data Sources, and Participants

This retrospective cohort study included patients from a large health system in New York (NYU Langone Health). Data were obtained through the electronic health record (EHR) (Epic Systems), which was linked to medication fill data from pharmacies and pharmacy benefit managers nationwide (Surescripts) and census-tract level data (US Census Bureau’s American Community Survey [ACS],^19^ NYC Department of Transportation Transit Travelshed Model,^20^ Walk Score ratings,^21^ and National Neighborhood Data Archive National Establishment Time Series^22^). Adults with the following were included: (1) HF diagnosis; (2) prior left ventricular ejection fraction (LVEF) of 40% or lower on echocardiogram; and (3) prescription for at least 1 GDMT between June 30, 2020, and June 30, 2021 (diagnosis codes and medications in the eMethods in Supplement 1). GDMT included the following classes, grouped into 4 categories: (1) BB; (2) ACEI, ARB, or ARNI; (3) MRA; or (4) SGLT2i. Patients with improved LVEF were included given recommendations to continue GDMT in this population.^23^ The study was approved by the NYU Grossman School of Medicine institutional review board and met criteria for a waiver of informed consent based on Federal Policy for the Protection of Human Subjects (45 CFR 46.116), including that the research involved no more than minimal risk to participants and could not be carried out practicably without the waiver. This report follows the Strengthening the Reporting of Observational Studies in Epidemiology (STROBE) reporting guideline.^24^

### Measures

#### Primary Exposure

To obtain nSES, we geocoded patient addresses (Geocodio)^25^ for linkage to the US Census Bureau’s ACS,^19^ using ACS data from 2015 to 2019 for primary analyses to ensure the exposure was measured prior to mediators and outcomes for mechanistic interpretation. ACS variables were used to compute the Agency for Healthcare Research and Quality SES score,^26^ which combined information on crowding, property value, unemployment, poverty level, income, and education. This score was categorized in quartiles, with the lowest quartile corresponding to lowest nSES. Patients with missing nSES (ie, due to PO box address or missing ACS variables) were included in models as a separate category to limit selection bias.^27^

#### Outcomes

Nonadherence to GDMT over 6 months was measured using the proportion of days covered (PDC) metric recommended by the National Quality Forum and Pharmacy Quality Alliance (PQA).^28,29^ As previously described by others,^30^ PDC was defined as the ratio of days a medication was filled to days a prescription was active; lapses in prescription were excluded from the denominator. Two data sources were used to compute PDC: (1) pharmacy fill information (Surescripts) and (2) prescription information (EHR). As previously described by our group,^31^ this EHR-linked data set was comprehensive and contained more prescription fill information than insurance claims data. PDC was measured over 6 months from time of prescription order and averaged across GDMT categories if more than 1 GDMT was prescribed, as previously described.^32^ Patients who died during the study were censored. Given our aim to study chronic GDMT, medication categories prescribed for fewer than 28 days were excluded from calculations. The primary outcome was binary: mean PDC less than 0.8 (nonadherent) and mean PDC greater than or equal to 0.8 (adherent). This classification is widely used in the literature, recommended by the PQA, and has previously been associated with rehospitalization and death among patients with HFrEF.^29,33^ PDC was also tabulated as a continuous outcome for descriptive purposes.

#### Demographic and Clinical Covariates

We obtained patient age, sex, race (categorized as Asian, Black, White, and other [American Indian or Alaska Native, Native Hawaiian or Other Pacific Islander]), ethnicity (Hispanic/Latinx, non-Hispanic/Latinx), preferred language (English, Spanish, other), and insurance (Medicare, Medicaid, Private, other) from the EHR. To assess clinical severity, we also obtained most recent LVEF (<25%, 26%-35%, 36%-40%, or >40%), any hospitalization in the prior year, any emergency department (ED) visit in the prior year, and Elixhauser comorbidity index.^34^

#### Neighborhood-Level Mediators

**Figure 1.**
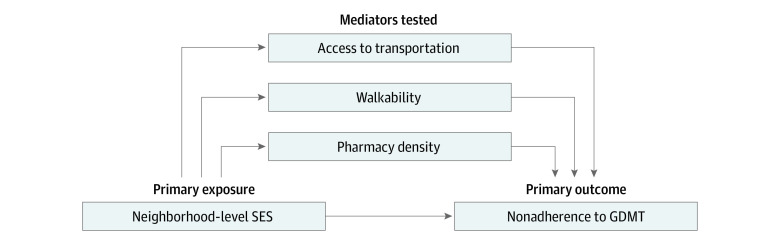
Direct Acyclic Graph Depicting Mediation Analyses GDMT indicates guideline-directed medical therapy; SES, socioeconomic status.

The following neighborhood-level mediators were assessed: access to transportation, walkability, and pharmacy density (direct acyclic graph in Figure 1 assumes causal relationships have no feedback). Access to transportation was measured using the 2018 NYC Transit Travelshed Model,^20^ which computes area reached within 60 minutes by walking, rail, subway, bus, and ferry. Walkability was assessed using the 2022 Walk Score,^21^ which rates ease of walking from 0 (lowest) to 100 (highest). Pharmacy density was obtained from the 2017 National Neighborhood Data Archive^22^ and was defined as number of pharmacies per 10 000 people.

### Statistical Analysis

Baseline characteristics were assessed across nSES categories with χ^2^ tests or analysis of variance (ANOVA) as appropriate. Multivariable logistic regression models were used to assess the association between nSES categories and odds of nonadherence, with and without adjustment for age, sex, most recent LVEF, comorbidity index, hospitalization in the past year, and ED visit in the past year. For interpretation, difference in probability of nonadherence between each nSES category and the highest quartile, averaged over covariates, was derived from the fitted logistic regression model and reported as a measure of total effect. We chose not to include individual patient race, ethnicity, language, and insurance status in multivariable models. This is because current and historical institutional and structural racism has led to racial and ethnic segregation among neighborhoods, and variations in health behavior by race, ethnicity, or language may, in part, be mediated by nSES factors.^35,36^ Moreover, these variables may be associated with individual-level SES factors, such as income, wealth, and education, which were not reliably available in our data set. Furthermore, associations between individual-level and neighborhood-level SES variables could be bidirectional and lead to a combination of mediation and/or confounding mechanisms.^37^ Inclusion of these variables without being able to adjust for these other associations could lead to biased or incomplete conclusions. Therefore, our primary analytic model aimed to assess nSES as a global risk factor for nonadherence, rather than attempting to isolate the independent effect of nSES from the myriad of measurable and unmeasurable individual-level factors.

In secondary stratified analyses, to evaluate for the specific intersectionality of nSES and race and ethnicity, we assessed interactions between race, ethnicity, and nSES. To do so, we used stratified multivariate logistic regression models similar to the primary analysis for the following subgroups: race (Black vs White) and ethnicity (Hispanic/Latinx vs non-Hispanic/Latinx). ANOVA test was used to assess *P *for interaction for adjusted models with and without interaction term for each subgroup.

Separate mediation analyses were conducted to estimate the extent to which nSES disparities in nonadherence were mediated through each built environment mediator. For each mediator, multivariable logistic regression models were used to assess the associations between (1) nSES and built environment factor (association of exposure with mediator) and (2) nSES and nonadherence (association of exposure with outcome), adjusting for the same covariates described previously. Estimated coefficients from the 2 models were combined to estimate natural average direct and natural average indirect effects as differences in probabilities of nonadherence. Bootstrapped confidence intervals with 1000 simulations were computed.^38^ Proportion mediated was reported when the indirect effect was significant and along the direction of the direct effect. Due to data availability, analysis for transportation was restricted to patients in New York City. Additionally, analysis for pharmacy density used 2013-2017 ACS data to allow for exposure prior to mediator. Analyses were conducted using R version 4.2.2 (R Project for Statistical Computing). Two-sided *P* < .05 indicated statistical significance.

## Results

### Baseline Characteristics

Among 6247 included patients, the mean (SD) age was 73 (14) years, and majority were male (4340 [69.5%]). There were 1011 (16.2%) Black participants, 735 (11.8%) Hispanic/Latinx participants, and 3929 (62.9%) White participants. Overall, 599 (9.6%) had missing nSES data, and remaining patients were evenly distributed across nSES quartiles, with 3555 (55.3%) living in New York City. Several baseline characteristics varied by nSES category (Table 1). Patients living in lower nSES areas tended to be younger; were more often Black or other race, Hispanic/Latinx ethnicity, Spanish-speaking; had Medicaid insurance, lower LVEF, and lower comorbidity index scores; more often visited the ED in the past year; and were more often prescribed ARNI. Access to transportation and pharmacy density were lower for patients in lower nSES areas, and walkability was higher.

**Table 1.** Baseline Characteristics by Neighborhood-Level Socioeconomic Status

Characteristic	Neighborhood-level socioeconomic status quartile, No. (%)					*P* value^a^
	1 (n = 1413)	2 (n = 1411)	3 (n = 1417)	4 (n = 1407)	Missing (n = 599)	
Individual-level variables						
Age, mean (SD), y	69.2 (15.0)	72.5 (13.8)	74.1 (13.0)	75.7 (13.1)	71.8 (14.0)	<.001
Sex						
Female	456 (32.3)	429 (30.4)	440 (31.1)	406 (28.9)	176 (29.4)	.35
Male	957 (67.7)	982 (69.6)	977 (68.9)	1001 (71.1)	423 (70.6)	
Race						
Asian	83 (5.9)	70 (5.0)	45 (3.2)	67 (4.8)	18 (3.0)	<.001
Black	398 (28.2)	274 (19.4)	134 (9.5)	80 (5.7)	125 (20.9)	
White	564 (39.9)	832 (59.0)	1084 (76.5)	1135 (80.7)	314 (52.4)	
Other^b^	292 (20.7)	171 (12.1)	117 (8.3)	80 (5.7)	110 (18.4)	
Refused, unknown, or missing	76 (5.4)	64 (4.5)	37 (2.6)	45 (3.2)	32 (5.3)	
Ethnicity						
Hispanic/Latinx	304 (21.5)	137 (9.7)	118 (8.3)	72 (5.1)	104 (17.4)	<.001
Non-Hispanic/Latinx	991 (70.1)	1151 (81.6)	1204 (85.0)	1210 (86.0)	452 (75.5)	
Refused, unknown, missing	118 (8.4)	123 (8.7)	95 (6.7)	125 (8.9)	43 (7.1)	
Language						
English	1026 (72.6)	1085 (76.9)	1228 (86.7)	1304 (92.7)	465 (77.6)	<.001
Spanish	173 (12.2)	58 (4.1)	49 (3.5)	27 (1.9)	51 (8.5)	
Other	210 (14.9)	265 (18.8)	137 (9.7)	70 (5.0)	81 (13.5)	
Missing	4 (0.3)	3 (0.2)	3 (0.2)	6 (0.4)	2 (0.3)	
Insurance						
Medicare	840 (59.4)	904 (64.1)	949 (67.0)	986 (70.1)	398 (66.4)	<.001
Medicaid	250 (17.7)	159 (11.3)	108 (7.6)	71 (5.0)	92 (15.4)	
Commercial	290 (20.5)	310 (22.0)	306 (21.6)	301 (21.4)	90 (15.0)	
Other or missing	33 (2.3)	38 (2.7)	54 (3.8)	49 (3.5)	19 (3.2)	
Most recent LVEF, %^c^						
<25	299 (21.2)	246 (17.4)	246 (17.4)	212 (15.1)	121 (20.2)	<.001
25-35	677 (47.9)	695 (49.3)	663 (46.8)	677 (48.1)	296 (49.4)	
36-40	319 (22.6)	346 (24.5)	390 (27.5)	366 (26.0)	122 (20.4)	
>40	118 (8.4)	124 (8.8)	118 (8.3)	152 (10.8)	60 (10.0)	
Comorbidity burden (Elixhauser Index), mean (SD)	13.3 (7.33)	13.8 (7.17)	13.6 (6.79)	14.4 (7.68)	13.5 (7.76)	<.001
ED visit, past year	235 (16.6)	179 (12.7)	159 (11.2)	184 (13.1)	98 (16.4)	<.001
Hospitalization, past year	512 (36.2)	482 (34.2)	501 (35.4)	492 (35.0)	220 (36.7)	.75
GDMT prescribed, mean (SD), No.	2.26 (0.911)	2.22 (0.885)	2.20 (0.871)	2.19 (0.877)	2.20 (0.902)	.26
GDMT classes prescribed						
BB	1305 (92.4)	1296 (91.8)	1322 (93.3)	1300 (92.4)	545 (91.0)	.42
ACEI/ARB	727 (51.5)	713 (50.5)	755 (53.3)	742 (52.7)	313 (52.3)	.62
ARNI	450 (31.8)	454 (32.2)	398 (28.1)	390 (27.7)	175 (29.2)	.02
MRA	513 (36.3)	507 (35.9)	476 (33.6)	489 (34.8)	216 (36.1)	.56
SGLT2i	200 (14.2)	163 (11.6)	173 (12.2)	164 (11.7)	66 (11.0)	.15
Neighborhood-level variables, mean (SD)						
Access to transportation^d^	39 000 (13 100)	36 600 (12 800)	37 400 (15 400)	60 600 (22 000)	46 100 (22 700)	<.001
Walkability^e^	74.0 (25.0)	71.0 (24.8)	63.8 (25.9)	65.2 (28.4)	83.0 (24.0)	<.001
Pharmacy density^f^	3.47 (5.10)	3.21 (4.15)	3.32 (0.348)	3.74 (4.13)	22.7 (295)	<.001

Abbreviations: ACEI, angiotensin converting enzyme inhibitor; ARB, angiotensin receptor blocker; ARNI, angiotensin receptor neprilysin inhibitor; BB, β-blocker; ED, emergency department; GDMT, guideline-directed medical therapy; LVEF, left ventricular ejection fraction; MRA, mineralocorticoid receptor antagonist; SGLT2i, sodium glucose cotransporter 2 inhibitor.

^a^ *P* values reflect unadjusted differences across the 5 neighborhood socioeconomic status categories using χ^2^ tests for proportions or analysis of variance for means as appropriate.

^b^ Other race included American Indian or Alaska Native, Asian, and Native Hawaiian or Other Pacific Islander.

^c^ Patients were required at least one LVEF≤40 for inclusion, but were still included if LVEF recovered.

^d^ Access to transportation was measured using the transportation mobility index, defined as the geographical area in acres able to be reached within 60 minutes of transit.

^e^ Walkability was measured using the Walk Score rating on a scale of 0 to 100, with higher score indicating greater ease of walking within a neighborhood.

^f^ Pharmacy density was measured as number of pharmacies per 10 000 people.

### Association Between nSES and Medication Nonadherence

The percentage of patients with nonadherence to GDMT (PDC <0.8) was higher among patients in lower nSES areas and missing nSES, with rates in quartiles 1 to 4 and missing of 51.7% (731 of 1086), 47.5% (670 of 1094), 41.5% (588 of 1077), 40.0% (563 of 1086), and 53.9% (323 of 599), respectively (*P* < .001) (Table 2). Mean (SD) PDC was also lower (indicating lower adherence) for those in lower nSES areas and missing nSES, with quartiles 1 to 4 and missing having mean (SD) PDC of 0.619 (0.319), 0.650 (0.380), 0.710 (0.352), 0.712 (0.361), and 0.614 (0.387), respectively (*P* < .001).

**Table 2.** Unadjusted Rates of Medication Nonadherence by Neighborhood-Level Socioeconomic Status

Outcome	Neighborhood-level socioeconomic status quartile					*P* value
	1 (n = 1086)	2 (n = 1094)	3 (n = 1077)	4 (n = 1086)	Missing (n = 599)	
Nonadherence to GDMT (PDC <0.8), No. (%)	731 (51.7)	670 (47.5)	588 (41.5)	563 (40.0)	323 (53.9)	<.001
Adherence to GDMT (PDC as a continuous outcome), mean (SD)	0.619 (0.391)	0.650 (0.380)	0.710 (0.352)	0.712 (0.361)	0.614 (0.387)	<.001

Abbreviations: GDMT, guideline directed medical therapy; PDC, proportion of days covered.

**Figure 2.**
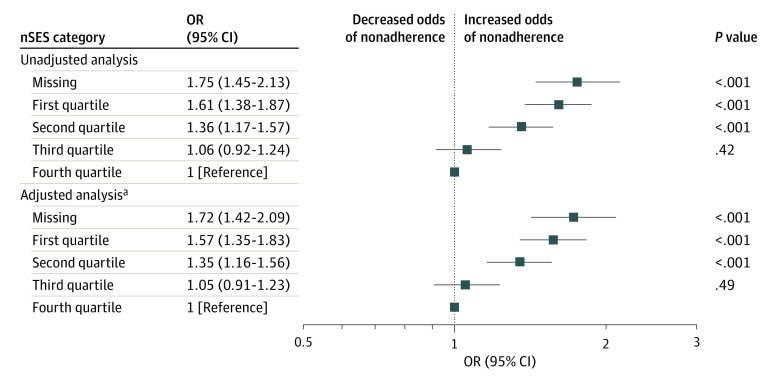
Odds Ratios (ORs) of Medication Fill Nonadherence to Guideline-Directed Medical Therapy by Neighborhood Socioeconomic Status (nSES) Quartiles

In unadjusted analysis, there was a graded association between nSES and nonadherence. When compared with patients in the highest nSES quartile, patients in the 2 lowest nSES quartiles had higher odds of nonadherence (quartile 1: odds ratio [OR], 1.61 [95% CI, 1.38-1.87]; *P* < .001; quartile 2: OR, 1.36 [95% CI, 1.17-1.57], *P* < .001) (Figure 2), while patients in the third quartile had similar odds of nonadherence (OR, 1.06 [95% CI, 0.92-1.24], *P* = .42). In adjusted analysis, this association persisted (quartile 1: OR, 1.57 [95% CI, 1.38-1.87]; *P* < .001; quartile 2: OR, 1.35 [95% CI, 1.16-1.56]; *P* < .001; quartile 3: OR, 1.05 [95% CI, 0.91-1.23]; *P* = .49). This corresponded to an 11.1% (95% CI, 7.5%-14.7%) and 7.2% (95% CI, 3.8%-10.9%) risk difference in probability of nonadherence between quartile 1 and quartile 4 and between quartile 2 and quartile 4, respectively (total effect, Table 3). Compared with patients in the highest nSES quartile, patients with missing nSES also had higher odds of nonadherence (unadjusted OR, 1.75 [95% CI, 1.45-2.13]; *P* < .001; adjusted OR, 1.72 [95% CI, 1.42-2.09]; *P* < .001).

**Table 3.** Estimated Differences in Probabilities of Nonadherence for Direct, Indirect, and Total Effects of the Association Between nSES and Medication Nonadherence and Percentage Mediated by Built Environment Factors^a^

nSES quartile	Natural direct effect (nSES to nonadherence), % (95% CI)^b^	Natural indirect effect (nSES to mediator to nonadherence), % (95% CI)^c^	Total effect (direct + indirect effect), % (95% CI)^d^	% Mediated
**Access to transportation**				
4	[Reference]	[Reference]	[Reference]	NA
3	1.0 (−4.9 to 6.3)	−0.5 (−2.3 to 1.4)	0.5 (−4.4 to 5.5)	NA
2	5.1 (0.1 to 10.1)^e^	−0.4 (−2.0 to 1.2)	4.7 (0.0 to 9.7)^e^	NA
1	7.6 (2.3 to 12.7)^f^	−0.4 (−1.9 to 1.1)	7.2 (2.1 to 12.2)^f^	NA
**Walkability**				
4	[Reference]	[Reference]	[Reference]	NA
3	1.4 (−2.2 to 5.0)	−0.1 (−0.3 to 0.7)	1.3 (−2.3 to 4.9)	NA
2	6.7 (3.3 to 10.3)^g^	0.5 (0.2 to 0.9)^g^	7.2 (3.8 to 10.9)^g^	6.9^g^
1	10.4 (6.6 to 14.1)^g^	0.7 (0.3 to 1.2)^g^	11.1 (7.5 to 14.7)^g^	6.5^g^
**Pharmacy density**				
4	[Reference]	[Reference]	[Reference]	NA
3	2.7 (−1.2 to 6.3)	0.0 (−0.1 to 0.2)	2.7 (−1.2 to 6.3)	NA
2	6.5 (3.0 to 10.1)^g^	0.0 (−0.1 to 0.1)	6.5 (3.0 to 10.0)^g^	NA
1	14.6 (10.8 to 18.0)^g^	0.0 (−0.1 to 0.1)	14.6 (10.8 to 18.0)^g^	NA

Abbreviations: NA, not applicable; nSES, neighborhood-level socioeconomic status.

^a^ Adjusted for age, sex, ejection fraction, emergency department visit in past year, hospitalization in past year, and Elixhauser comorbidity index.

^b^ Natural direct effect is the estimated difference in rates of nonadherence independent of the built environment factor for patients in 1 nSES quartile when compared with the highest nSES quartile, ie, the difference in risk if the built environment factor was the same in both nSES quartiles.

^c^ Natural indirect effect is the estimated difference in rates of nonadherence transmitted through the built environment factor for patients in 1 nSES quartile compared with the highest nSES quartile, ie, the difference in risk that is changing due to changes in the built environment factor.

^d^ Total effect is the sum of the direct and indirect effects. Note, total effects are different for the different mediation analyses due to differences in cohort (New York City–only cohort for access to transportation) and exposure (2013-2017 nSES calculation for pharmacy density).

^e^ *P* ≤ .05.

^f^ *P* < .01.

^g^ *P* < .001.

### Interaction and Mediation Analyses

There was no significant interaction between race or ethnicity on the association between nSES and odds of nonadherence (eFigure in Supplement 1). There was no significant indirect effect (ie, effect through the mediator) observed for access to transportation or pharmacy density (Table 3). For walkability, a small, but statistically significant indirect effect was observed, such that walkability contributed to approximately 7% of the variability in probability of nonadherence for patients in nSES quartiles 1 or 2 compared with quartile 4 (percentage mediated, *P* < .001). In absolute terms, higher walkability appeared to be protective in quartiles 1 and 2 compared with quartile 4 and contributed to an estimated improvement in nonadherence by 0.7% and 0.5%, respectively (indirect effects, *P* < .001).

## Discussion

In this retrospective cohort study of patients with HFrEF, living in a lower nSES area was associated with higher odds of nonadherence to GDMT as measured by pharmacy fills. This association persisted after adjustment for clinical covariates. Our findings highlight an important inequity in HFrEF treatment, reveal a key risk factor for medication nonadherence, and underscore the importance of considering neighborhood-level disparities when developing initiatives to improve medication adherence.

Patients with HFrEF are known to have considerable shortfalls in medication adherence that contribute to increased mortality and hospitalization.^6^ Our findings inform the hypothesis that neighborhood-level disparities contribute to these shortfalls. Overall, nonadherence to GDMT was common in our cohort, occurring in 52% of patients in the lowest nSES quartile and 40% of patients in the highest nSES quartile. This range of nonadherence is consistent with other studies in HF,^3,4,5,6,39^ further confirming the extent of this problem. Moreover, the magnitude of the disparity in nonadherence by nSES that we observed (risk difference >10%) was likely clinically meaningful, given that decreases in GDMT adherence by even 5% to 10% are associated with significant increases in mortality among patients with HF.^6,39^ Given that patients with HFrEF living in lower nSES areas tend to have increased mortality and readmission,^40^ understanding and mitigating barriers to medication adherence in this population has the potential to ultimately reduce inequities in clinical outcomes.

Our finding of higher rates of medication nonadherence for patients with HFrEF living in lower nSES neighborhoods was consistent with observations in other diseases or conditions, including cardiovascular disease prevention, diabetes, and chronic kidney disease.^7,8,9,41^ Our study also addressed limitations of prior work that utilized administrative claims data,^8,9^ which are often inherently restricted to a single insurance type; lack the ability to incorporate clinical covariates, such as LVEF; and are unable to account for prescribing actions.

Overall, the association between nSES and medication nonadherence is likely multifaceted and due to complex, dynamic interactions between environmental, individual, and cognitive factors.^42^ Neighborhood-level factors include environmental stressors, such as violence and perceived safety, which could serve as physical barriers^43^ to obtaining medications, and also act as emotional stressors that drain one’s capacity for adherence in the setting of immediate threat.^12^ Additionally, built environment factors, such as access to transportation, walkability, and access to health services, could also impede one’s ability to physically obtain medications, or could impede interactions with health care professionals to discuss medication-related concerns.^44^ Moreover, neighborhood-level social factors contributing to medication nonadherence could include social cohesion and social norms about medications.^12,45^ Beyond neighborhood-level factors, associated individual-level factors could include medication cost, income, family support, health literacy, health care interactions, language, and individual beliefs.^12^ For example, several studies in HF have shown an association between income,^15^ medication costs,^14,46,47,48^ and nonadherence to GDMT, most recently for the newer classes, ARNI^14,15^ and SGLT2i.^13^ In our study, patients living in lower nSES areas may have had lower income, which could make them more vulnerable to medication nonadherence.

In our mediation analysis, we had hypothesized that built environment barriers to physically obtaining medications would partly explain neighborhood-level disparities in nonadherence. Surprisingly, we found no mediation by access to transportation or pharmacy density, and only a small mediation effect by walkability. This suggests that the association between nSES and medication nonadherence is more likely due to the myriad of other environmental and individual factors contributing to medication nonadherence discussed previously. It is also likely that the built environment measures that we used did not capture the complex manner in which patients obtain medications. For example, disparities in pharmacy accessibility can go beyond location, and can include disparities in hours of operation, availability and price of medications, engagement with pharmacists, and discrimination by staff.^17,49,50,51^ Similarly, our transportation and walkability indices did not capture nuances of individual-level distances, private vehicle ownership, or drive-through options. Moreover, transportation barriers in urban and suburban environments likely differ, and our observation of higher walkability in lower nSES neighborhoods may reflect this. Finally, use of mail-order services could mitigate built environment barriers, and we did not assess this. Therefore, although increasing pharmacies, adding transportation routes, and improving walkability likely has benefits, these efforts are probably insufficient to address nSES disparities in medication nonadherence.

Several team-based interventions have been shown to improve medication adherence and reduce mortality and hospitalization for patients with HF.^52^ These often involve patient education, monitoring, and frequent follow-up with health professionals. Our findings support the adaptation of these interventions for patients living in at-risk neighborhoods. For example, involving community health workers and local pharmacists in such programs could help patients overcome distrust, link patients to community resources, and provide up-front face-to-face counseling.^53,54,55,56^ Additionally, screening for neighborhood-level risk in clinical practice^57^ could provide opportunities to match patients to local programs.

### Limitations

This was a retrospective cohort study conducted across a single health system in an urban environment. PDC measures medication adherence through pharmacy fills, which may not reflect what the patient is actually taking. We chose a PDC threshold of less than 0.8 based on previously described associations with adverse outcomes in HFrEF,^33^ but the optimal PDC threshold for this population is unknown.^58^ We did not assess whether prescriptions were filled by mail. Due to the nature of EHR data, we were unable to reliably account for time since HF diagnosis or HF treatment history. The relationship between neighborhood-level and individual-level factors is complex,^37^ and due to our limited EHR data on individual-level SES variables, we chose to assess nSES as a global risk factor. *P* values for interaction and mediation analyses were not adjusted for multiple testing and should be interpreted cautiously. Of note, we found no statistically significant interaction between individual race or ethnicity on the association between nSES and nonadherence, which may have been due to the relatively small proportion of Black and Hispanic/Latinx patients in our sample. Although we accounted for temporality by using primary exposure data that was collected prior to mediators and outcomes, we could not account for neighborhoods changing over time, patients moving between neighborhoods, or patients leaving the area. Walkability data were obtained 1 year after the study period, although it is unlikely to have changed substantially over a year. We included patients with missing nSES as a separate exposure category to reduce selection bias, as previously described.^27^ Interpretation of associations between missing nSES and nonadherence is limited due to incompletely understood reasons for missing data. While our approach was based on a directed acyclic graph guided by the theoretical framework to assess mechanistic associations, important confounders may be unmeasured or missing.

## Conclusions

We found higher rates of nonadherence to GDMT among patients with HFrEF living in lower nSES areas. This association remained after adjustment for clinical characteristics. Our findings highlight an important gap that may contribute to known neighborhood-level disparities in HF care and outcomes. Future investigations and initiatives should focus on identifying neighborhood-level barriers to adherence, targeting interventions to neighborhoods at highest risk, and adapting them to address community needs.

## Notes

Supplement 1. **eMethods.** Detailed Methods. **eFigure.** Stratified Analysis

Supplement 2.
Data Sharing Statement
