# Supplementary material for: Neighborhood-Level Socioeconomic Status and Prescription Fill Patterns Among Patients With Heart Failure
Source: JAMA Netw Open. 2023 Dec 14;6(12):e2347519. doi: 10.1001/jamanetworkopen.2023.47519 (PMC10722333; doi:10.1001/jamanetworkopen.2023.47519)
Supplement: Supplement 1. — eMethods. Detailed Methods. eFigure. Stratified Analysis [file jamanetwopen-e2347519-s001.pdf]

Supplemental Online Content

Mukhopadhyay A, Blecker S, Li X, et al. Neighborhood-level socioeconomic status and prescription fill patterns among patients with heart failure. *JAMA Netw Open.* 2023;6(12):e2347519. doi:10.1001/jamanetworkopen.2023.47519

eMethods. Detailed Methods.

eFigure. Stratified Analysis

This supplemental material has been provided by the authors to give readers additional information about their work.

© 2023 Mukhopadhyay A et al. *JAMA Network Open.*

eMethods. Detailed Methods.

ICD-10 diagnoses codes: The following diagnoses codes were used for inclusion into the study, selected using methodology from Center for Medicare & Medicaid Services.[59] For inclusion, diagnoses were listed in the problem list, visit diagnosis, or billing code.

1. I50.x Heart Failure
2. I11.0 Hypertensive heart disease with heart failure
3. I13.0 Hypertensive heart and chronic kidney disease with heart failure and stage 1 through stage 4 chronic kidney disease, or unspecified chronic kidney disease
4. I13.2 Hypertensive heart and chronic kidney disease with heart failure and with stage 5 chronic kidney disease, or end stage renal disease

Full list of medications included as GDMT: The following medications were considered acceptable for each medication class (note, long-acting, short-acting, brand-name, and combination-pill formulations were all included).

1. BB – bisoprolol, carvedilol, metoprolol (succinate or tartrate)
2. ACEI – benazepril, captopril, enalapril, enalaprilat, fosinopril, lisinopril, moexipril, perindopril, quinapril, ramipril, trandolapril
3. ARB – azilsartan, candesartan, eprosartan, irbesartan, losartan, olmesartan, telmisartan, valsartan
4. ARNI – sacubritil/valsartan
5. MRA – epleronone, spironolactone
6. SGLT2i – empagliflozin, dapagliflozin

© 2023 Mukhopadhyay A et al. *JAMA Network Open.*

eFigure 1. Stratified Analysis

Association between nSES and medication non-adherence (PDC < 0.8) to GDMT, stratified by (A) race and (B) ethnicity.

Subgroup	nSES Category	N	OR (95% CI)	P-interaction
**A. PATIENT RACE**				
**Black**	Missing	125	1.49 (0.83-2.67)	
(N = 1011)	1st Quartile	398	1.05 (0.64-1.72)	
	2nd Quartile	274	1.03 (0.62-1.73)	
	3rd Quartile	134	1.01 (0.57-1.78)	
	4th Quartile	80	Reference	0.671
**White**	Missing	314	1.57 (1.22-2.03)	
(N = 3929)	1st Quartile	564	1.48 (1.20-1.81)	
	2nd Quartile	832	1.33 (1.11-1.60)	
	3rd Quartile	1084	1.13 (0.95-1.34)	
	4th Quartile	1135	Reference	
**B. PATIENT ETHNICITY**				
**Hispanic/Latinx**	Missing	104	1.53 (0.83-2.83)	
(N = 735)	1st Quartile	304	1.09 (0.64-1.84)	
	2nd Quartile	137	1.31 (0.73-2.35)	
	3rd Quartile	118	0.82 (0.45-1.49)	
	4th Quartile	72	Reference	0.229
**Non-Hispanic/Latinx**	Missing	452	1.79 (1.44-2.23)	
(N = 5008)	1st Quartile	991	1.74 (1.46-2.07)	
	2nd Quartile	1151	1.37 (1.16-1.61)	
	3rd Quartile	1204	1.11 (0.94-1.31)	
	4th Quartile	1210	Reference	

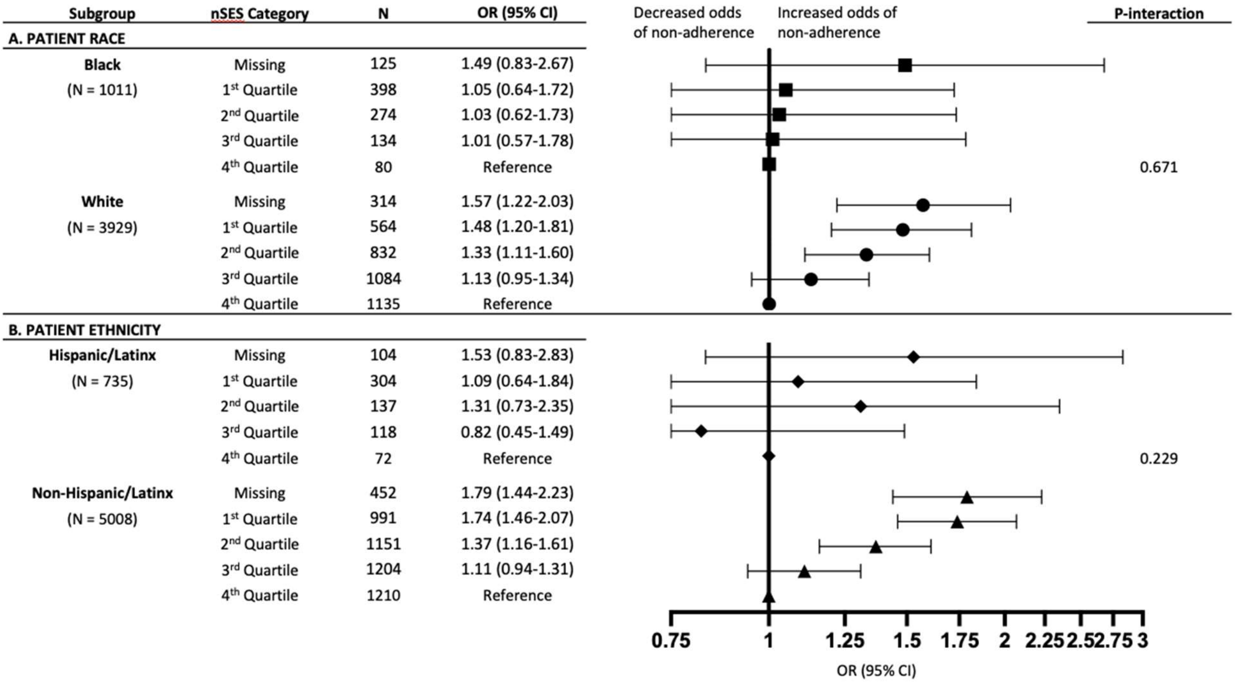

Each analysis is adjusted for age, sex, ejection fraction, emergency department visit in past year, hospitalization in past year, and Elixhauser comorbidity index. nSES – neighborhood level socioeconomic status; GDMT – Guideline-directed medical therapy; PDC – proportion of days covered; OR – Odds ratio; CI – Confidence interval

© 2023 Mukhopadhyay A et al. *JAMA Network Open.*
