# Supplementary material for: Neighborhood-Level Socioeconomic Status and Prescription Fill Patterns Among Patients With Heart Failure
Source: JAMA Netw Open. 2023 Dec 14;6(12):e2347519. doi: 10.1001/jamanetworkopen.2023.47519 (PMC10722333; doi:10.1001/jamanetworkopen.2023.47519)
Supplement: Supplement 2. — Data Sharing Statement [file jamanetwopen-e2347519-s002.pdf]

# Data Sharing Statement

Mukhopadhyay. Neighborhood-Level Socioeconomic Status and Prescription Fill Patterns Among Patients With Heart Failure. *JAMA Netw Open*. Published December 14, 2023. doi:10.1001/jamanetworkopen.2023.47519

**Data**
**Data available:** No
